# The impact of learning styles on attitudes to interprofessional learning among nursing students: a longitudinal mixed methods study

**DOI:** 10.1186/s12912-023-01225-9

**Published:** 2023-03-13

**Authors:** Susanne Lundell Rudberg, Hanna Lachmann, Taina Sormunen, Max Scheja, Margareta Westerbotn

**Affiliations:** 1 Department of Learning, Informatics, Management and Ethics, Karolinska Institutet, 171 77 Stockholm, Sweden; 2 Department of Health Promoting Science, Sophiahemmet University, P. O. Box 5605, 114 86 Stockholm, Sweden; 3 Department of Education of Stockholm University, 106 91 Stockholm, Sweden; 4 Department of Nursing Science, Sophiahemmet University, P. O. Box 5605, 114 86 Stockholm, Sweden; 5 Department of Clinical Science and Education, Södersjukhuset, Karolinska Institutet, 118 83 Stockholm, Sweden

**Keywords:** Attitude, Collaborative learning, Interprofessional learning, Learning styles, Nursing Education Research, RIPLS, Students, nursing

## Abstract

**Background:**

A functional interprofessional teamwork improves collaborative patient-centred care. Participation in interprofessional education promotes cooperation after graduation. Individuals tend to use different approaches to learning depending on their individual preferences. The purpose of this study was to explore nursing students’ experiences of professional development with a focus on the relationship between attitudes to interprofessional learning and learning styles.

**Methods:**

A longitudinal parallel mixed-methods design. The study was carried out at a Swedish three-year nursing program from August 2015 to January 2020. On enrolment, thirty-four students self-assessed their attitudes to interprofessional learning according to the Readiness for Interprofessional Learning Scale, and their learning style according to Kolbs’ Learning Style Inventory. In the final semester the students participated in an interview focusing on their experiences and perceptions of teamwork and they self-assessed their attitudes to interprofessional learning again.

**Results:**

Our findings indicated that 64.7% had a predominantly concrete learning style and 35.3% had a predominantly reflective learning style. No significant relationship with internal consistency reliability was identified among the participants between attitudes to interprofessional learning and learning styles. The content analysis resulted in four main categories: *Amazing when it’s functional; Deepened insight of care; Increased quality of care; Understanding own profession* which were summarized in the theme: *Well-functioning teams improve patients’ outcome and working environment.*

**Conclusion:**

The students’ attitudes to interprofessional learning were positive and it was considered as an opportunity to participate in interprofessional cooperation during internship. Transformative learning is a useful strategy in fostering interprofessional relationships due to the interdependence of various professions in interprofessional teams. When students are guided to use reflection to develop new perspectives and meaning structures, they acquire emotional and rational skills beneficial for interprofessional cooperation.

**Supplementary Information:**

The online version contains supplementary material available at 10.1186/s12912-023-01225-9.

## Background

Nursing education focuses on professional as well as academic skills, aiming to train competent nurses who can deal with the complexity of modern health-care provision [1]. On entering the nursing program, students are often full of enthusiasm and eagerness to learn [2, 3].

### Learning styles

Depending on individual preferences, individuals tend to use different approaches to learning, so-called learning styles. A concrete learning style is characterized by an active, concrete, explorative and experimental approach [4]. The concrete learner uses practical application, focuses on feasibility, usability, utility, and results; a pragmatic approach guides the learning process. The reflective learning style is associated with emotional involvement, intuition, based own experiences and takes place in dialogue and collaboration with others [4]. According to Kolb’s Experiential Learning Theory (ELT) [4, 5], learning can be seen as an endless cycle of four stages. The ELT is a holistic theory of learning based on a learning cycle that defines learning as a fundamental process of human adaptation, driven by the resolution of the dual dialectics of action/reflection and experience/abstraction [6]. Observations and concrete experiences form the basis for reflection. When the individual reflects on immediate and concrete experiences, the construction of a general theory about the meaning of the information is initiated. The learner then creates generalizations based on the hypothesis using abstract concepts. In the fourth and final stage, the learner tests implications of these concepts in similar situations [4]. Kolb’s Learning Style Inventory (LSI) is a well-known and widely used instrument and has been frequently used in nursing education research. The classification of learning styles is based on individuals’ preferred approaches to acquiring knowledge trough concrete experience, abstract conceptualization, reflective observation and active experimentation [6]. In view of the LSI learners can be categorized into four learning styles: Accommodator, Diverger, Assimilator and Converger [4]. Accommodators actively engage in new experiences, implement their plans, adapt, and perform well under changed conditions. Divergers have an ability to see concrete situations from different perspectives, are creative and show an awareness of meaningful values. Assimilators reason inductively and create theoretical ideas. Convergers reason deductively, apply practical ideas, and perform well when there is an answer to a problem. Nursing students are commonly described in research as concrete and linear thinkers focussing on facts, preferring pictures, diagrams, flow charts and enjoying working in groups to try out different solutions to problems [7]. Learning activities based on learning styles has been found to facilitate the education of professional nurses, but there is a need for more research in this area [8].

### Interprofessional collaboration

Interprofessional collaboration (IPC), is considered to be an effective care model bridging task-related gaps that require efforts by various healthcare professionals [9]. Both international organisations and key agencies agree that IPC competencies are a key aspect for future health care workforce [10, 11]. The experiential learning in clinical setting has as its ambition to provide students with the opportunity of integrating theoretical understanding provided by academic courses with skills and knowledge acquired in practical settings. Part of the training in the clinical context involves practising collaboration both within and between professions [12]. Interprofessional Learning (IPL), promotes cooperation between students in two or more healthcare professions, for example between medical students and nursing students; thus students develop skills in communication and understanding of roles, resulting in improved collaborative patient-centred care [10, 13]. Supervisors support IPC and IPL, but there are challenges for implementation in clinical settings [14]. In terms of practical and logistical challenges, for instance, there may be a lack of space and encounters with vulnerable patients may require a limited number of caregivers [15]. Furthermore, young healthcare professionals appears to have positive attitudes towards interprofessional collaboration and undergraduate nursing students have reported a more positive attitude to IPL than have medical students [16, 17]. A previous Swedish study found no correlation between learning styles and attitudes towards interprofessional teamwork among medical students suggesting that further research could benefit from a combination of quantitative and qualitative research methods [18]. Research investigating the influence of learning styles on attitudes towards IPE and interprofessional collaboration is scares. To the best of the authors’ knowledge, there are no published studies exploring the correlation between learning styles and attitudes towards IPE among nursing students using a mixed method approach.

## Methods

### Aim

To explore nursing students’ experiences of professional development with a focus on the relationship between attitudes to IPL and learning styles.

### Design

A longitudinal parallel mixed-methods study design [19] was used to obtain different perspectives and build a comprehensive understanding of students’ attitudes to IPL and learning styles.

### Settings

This study took place at a Swedish university from August 2015 – January 2020. The nursing program followed the national guidelines, a three-year program (equivalent to 180 credits, according to the European Credit Transfer and Accumulation System, ECTS), of which clinical practice accounted for 60 ECTS. The main subject, nursing science, corresponded to 109 ECTS credits and medical science 71 ECTS credits. The program led to a professional degree as a Registered Nurse (RN), as well as a bachelor’s degree. The nursing program in Sweden is free of charge for citizens from Sweden, EU, EEA, and Switzerland. The first year consisted of theoretical training. During the second year of the education, approximately one third was made up of clinical training in elderly and medical care. The third year consisted mainly of clinical training within psychiatric, palliative, and primary health care, except for writing a bachelor’s thesis in the fifth semester. In the final semester students did their clinical training in advanced medical care, and in surgical care including two weeks at a clinical interprofessional training ward.

### Participants demographics

At the time of the interviews the mean age was 32.9 ± 9.1, ranged 22–53 and 83.2% (n = 28) were women and 17.6% (n = 6) were men. All students were Swedish citizens, 8.8% (n = 3) was born abroad and 20.6% (n = 7) students had parents from other countries. 41% reported studying fulltime, while 35.2% (n = 12) planned to work for salary at a maximum 10 h/week and 23.5%s (n = 8) planned to work 10–20 h/week. A total of 20.5% (n = 7) reported having an RN in the family while 44.1% (n = 15) had family members working in other professions in health care. 47% (n = 16) reported having previously attended higher education and 29.4% (n = 10) stated having completed a university degree in another subject.

## Data collection

All students enrolled were invited to participate during the first week of education. Information about the purpose of the study was given orally after an introductory lecture and in writing on the university’s learning platform. Students who signed a written informed consent were included in the study. All collected data were anonymized and coded before processing and were stored on a hard drive secured with password only accessible to the research team.

### Collection of quantitative data

Questionnaires were distributed via the university’s learning platform at the start of the program and at the start of the final semester. The first questionnaire included demographics, the Swedish version of the LSI [20] together with the Swedish version of the Readiness for Interprofessional Learning Scale (RIPLS) [21]. The second questionnaire included the Swedish version of the RIPLS [21]. RIPLS is a well-known and widely used questionnaire [22] that has been translated into many languages, including Swedish [21]. RIPLS builds on 19 questions concerning attitudes on interprofessional collaboration that generate four subscales. Higher scores indicate a more positive attitude towards interprofessional education [21, 22]. A 7-point Likert scale (1 corresponding to strongly disagree and 7 to strongly agree) were used for all questions instead of the original 5-point Likert scale [23].

### Collection of qualitative data

Towards the end of the final semester, participating students were invited to an individual, semi-structured interview carried out in an undisturbed room at the university. Students were also informed about whom to contact if they had questions prior to the interviews. A semi-structured interview-guide was developed to capture students’ attitudes to interprofessional collaboration. The interview-guide included the following questions:

- How do you perceive the nurse’s role in the interprofessional teamwork?
- What do you experience the nurse’s role to be, as perceived by others in the interprofessional team?
- What are your experiences of interprofessional teamwork and the role of the nurse? Positive, negative experiences?
- Has the interprofessional teamwork affected you during your education and, if so, how?
- Now that we have talked about your role as a nurse and your own experiences and experiences of interprofessional collaboration, is there anything you would like to add about these issues?

All interviews were carried out by the first author and lasted 30 min on average, until all questions had been covered and the student declared there was nothing more to add. The interviews were audio recorded and transcribed verbatim [24, 25].

## Data analysis

Analysis was performed based the data from the 34 students who completed the RIPLS questionnaires on both occasions and participated in the interview at the end of the program.

### Quantitative data

Questionnaires were analysed using The Statistical Package for Social Sciences, SPSS [26]. Calculation of the LSI scores was performed according to the model defined by Marke and Cesarec [20]. Sample percentages were calculated for learning styles. Mean scores and standard deviations (SD) were calculated for variables of the RIPLS at the start and in semester six. Scoring adhered to the convention used in RIPLS translation by reverse-scoring items 10, 11, and 12. Results are reported for the Swedish version of the RIPLS [21]. This scale is divided into four subscales; Teamwork and collaboration, item 1–9); Negative Professional identity, item 10–12; Positive Professional identity, item 13–16; Roles and responsibility item (17–19). To validate internal consistency reliability of the RIPLS four subscales Cronbach’s alfa was defined. Paired T-test was used to compare the RIPLS at the start with result in semester six in the whole sample and according to learning style. A p-value lower than 0.05 was regarded as statistically significant in present study.

### Qualitative data

The interview transcripts were read several times to make sense of the data as whole [27] and then analysed using qualitative content analysis with a manifest inductive approach [28], using NVivo software [29]. Identified codes were grouped into sub-categories labelled with a phrase that described the meaning content. Sub-categories were grouped by contextual meaning resulting in four main categories. From this categorization, an abstraction was derived describing an overarching theme [28, 30], Table 1. During the analysis, the findings were discussed within the research group, until consensus was established.

**Table 1:** Examples of the content analysis process

Meaning unit	Condensed meaning unit	Code	Subcategory	Main category
I absolutely think the best care for the patient is when everyone is working towards the same goal and everyone is like, you’re good at this, I’m good at this, we put it together, that’s how it should be for all patients	Best care when everyone does what they are good at	Best care of patients	Patients benefits	Increased quality of care
After all, you (RN) have a coordinating role and I really feel that when I’m out on internship, I think if the nurse wasn’t there it would be more of a chaos, so you have an extremely important function in coordinating and conducting dialogue with colleagues and to advocate for the patient’s case within the team	Very important function in coordinating and conducting dialogue with colleagues and taking the patient’s case further within the team.	Coordinate communication with other professions and patients	Communication expert	Understanding own profession

## Results

### Quantitative findings

**Fig. 1:**
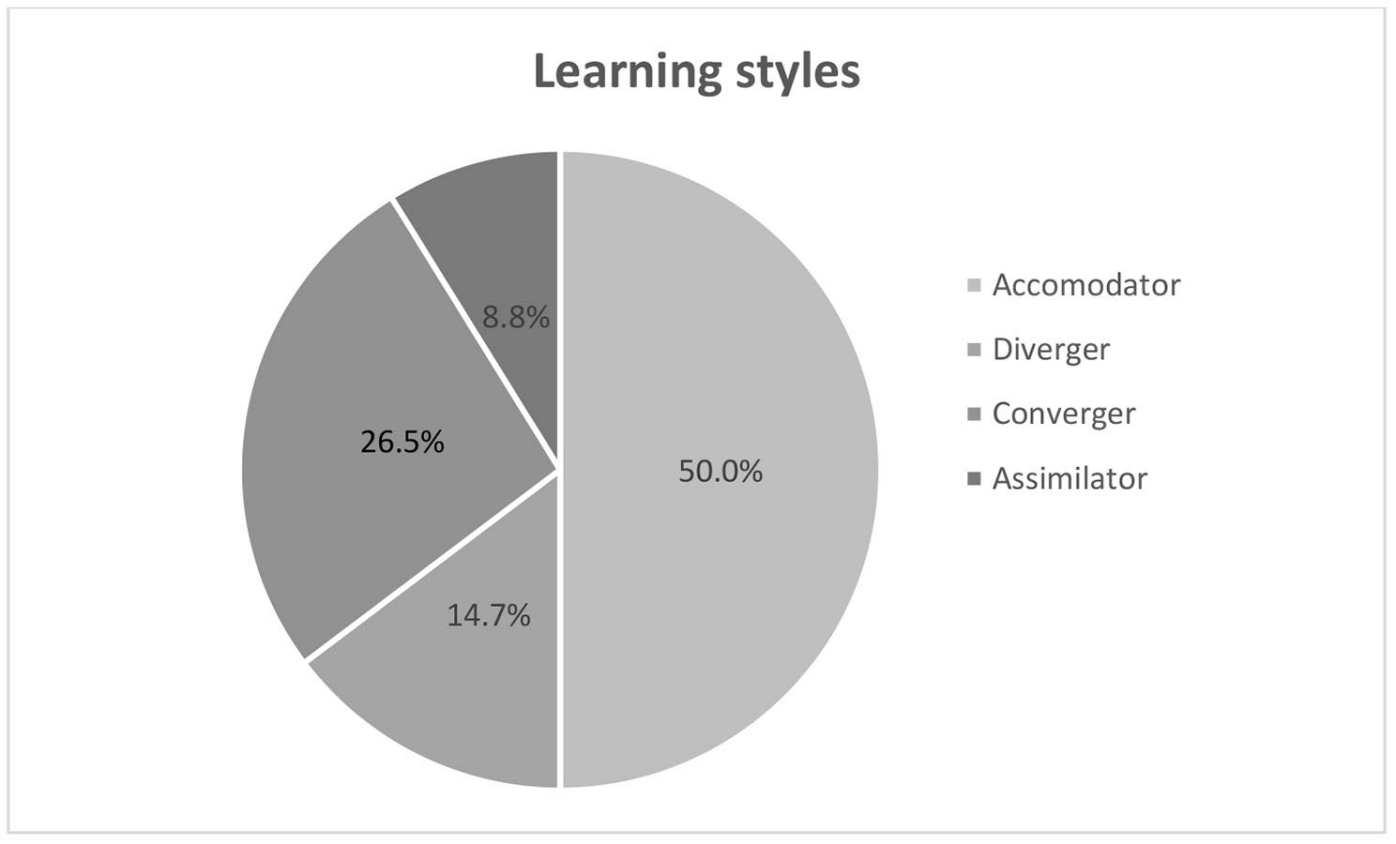
Distribution of the respondents’ (n = 34) learning styles in percent according to Kolb’s LSI.

Figure 1 shows the distribution of learning styles among respondents indicating that 64.7% had a predominantly concrete learning style (Accommodator 50% + Diverger 14.7%). Further, 35.3% were predominantly reflective (Assimilator 8.8% + Converger 26.5%).

Table 2 summarizes the participants’ attitudes to interprofessional learning at the start and in the final semester in a paired T-test. There was a significant difference between results at the start and the end of the education of the subscale Roles and responsibilities (p. < 000).

**Table 2:** Attitudes to interprofessional learning at the start of the education and in semester six (n = 34)

RIPLS subscales	At start			Semester 6			p-value
	Mean	StD	(α)	Mean	StD	(α)	
Teamwork and collaboration item 1–9	53.85	6.91	0.80	53.56	7.35	0.89	0.851
Negative Professional identity item 10–12	6.18	3.53	0.76	5.38	2.36	0.77	0.149
Positive Professional identity item 13–16	21.24	3.89	0.66	21.62	5.76	0.94	0.728
Roles and responsibility item 17–19	10.21	2.95	0.05	7.53	3.39	0.44	< 0.000*

StD Standard deviation

(α) Cronbach’s alpha

*Statistically significant, p-value < 0.5

Table 3 shows the difference in attitudes to interprofessional learning connected to learning style. There were significant changes in the RIPLS subscales Roles and responsibility in Accommodators (p = .006) and Convergers (p=. 003). No other relationship was found between the RIPLS and the LSI in performed analysis.

**Table 3:** Learning style and changes in RIPLS at the start and in semester six (n = 34)

RIPLS subscale	Learning style	Mean	StD	t	df	p-value
Teamwork and collaboration item 1–9	Accommodator (n = 17)	0.41	8.49	0.200	16	0.844
	Diverger (n = 5)	4.60	10.41	0.988	4	0.379
	Converger (n = 9)	-3.00	10.52	-0.855	8	0.417
	Assimilator (n = 3)	2.33	4.51	0.896	2	0.465
Negative Professional identity item 10–12	Accommodator (n = 17)	0.71	2.52	-1.155	16	0.265
	Diverger (n = 5)	2.20	6.06	0.812	4	0.462
	Converger (n = 9)	0.55	2.51	0.665	8	0.525
	Assimilator (n = 3)	-0.33	2.31	-0.250	2	0.826
Positive Professional identity item 13–16	Accommodator (n = 17)	-0.12	5.46	-0.089	16	0.930
	Diverger (n = 5)	-3.40	9.24	-0.823	4	0.457
	Converger (n = 9)	-0.56	7.09	-0.235	8	0.820
	Assimilator (n = 3)	3.67	3.22	1.976	2	0.187
Roles and responsibility item 17–19	Accommodator (n = 17)	2.47	3.20	3.179	16	.006*
	Diverger (n = 5)	2.20	3.83	1.283	4	0.269
	Converger (n = 9)	4.56	3.25	4.212	8	.003*
	Assimilator (n = 3)	-1.00	5.00	-0.346	2	0.762

StD Standard deviation

t t-value

df Degrees of freedom

* Statistically significant, p-value < 0.5

### Qualitative findings

Table 4 displays sub-categories, main categories, and theme of the content analysis of students’ attitudes to, and experiences of, teamwork.

**Table 4:** Content analysis of students’ attitudes to, and experiences of, interprofessional teamwork (n = 34)

Sub-categories	Main categories	Theme
Varying functionality	Amazing when it’s functional	Well-functioning teams improve patients’ outcome and working environment
Personality influences		
RN, the slop bucket		
Need more practice		
Alone isn’t strong	Deepened insight of care	
Developing with other professions		
Understanding competences		
Understanding complexity		
Common objective	Increased quality of care	
Contribute to the teamwork		
Patients benefits		
Teamwork improves communication		
Being listened to	Understanding own profession	
Communication expert		
Clarifies RN:s responsibilities		
Team leader, spider in the web		

The content analysis resulted in four main categories: (1) Amazing when it’s functional; (2) Deepened insight of care; (3) Increased quality of care; (4) Understanding own profession; were summarized in the theme: *Well-functioning teams improve patients’ outcome and working environment.*

#### Amazing when it’s functional

A functional interprofessional team was seen as an asset, both for work-place wellbeing and efficiency. Students highlighted the problems and obstacles that occur when teamwork is insufficient. The observations students made of the interprofessional theme were compared with their own experiences during IPL. The students underscored that IPE created good conditions for future cooperation with other professionals.

When it works, it’s fantastic… sometimes it feels like it’s getting a bit fragmented... and then it becomes like some people work here and then some people work there, you can’t coordinate that and it sort of falters, but I think that it is extremely important that it works

*(Student no. 34)*

#### Deepened insight of care

The students’ understanding of the care structure increased through interprofessional collaboration. Being tutored individually in the team was considered educational, however IPL was perceived as even more developmental. By communicating with students from other professions, the students increased their knowledge of more dimensions of patients’ needs for care and treatment.

That you get to work with other students and see what their attitudes were and then you also got to become a little more, you talked more about how to work around the patient

*(Student no. 16)*

#### Increased quality of care

Learning together with students from other professions was considered to benefit patient care. The students reflected on their own experiences of functioning and dysfunctional teamwork in clinical practice. It was emphasized that when everyone was familiar with the roles and responsibilities of other team members the quality of nursing, medical care and paramedicine improved.

I believe that as long as everyone knows their role and what they can contribute with and what everyone can contribute, we can provide better care to patients

*(Student no. 27)*

#### Understanding own profession

The students’ perception of the nurse’s role became clearer when they observed and participated in IPC. During IPL at student wards, students trained communication with other professions and practised the coordinating and leading role of the interprofessional team. Through collaboration with students from other professions, the nurse’s overall responsibility for nursing was clarified.

The spider in the web… all these clinical experiences you’ve gained in internships and things like that … you become the link between the doctors and the nurses, as if you end up somewhere in between, also with the occupational therapists and physiotherapists as well”

*(Student no. 30)*

### Correlation between quantitative and qualitative results

No statistically significant correlations were found between learning style and attitudes to interprofessional learning. There was a statistical difference between the two measurement occasions in the RIPLS subscale Roles and Responsibilities among Accommodators and Divergers, but no internal consistency reliability could be verified. Although there were no significant differences, the results from the content analysis indicate that IPL is considered educational and that *well-functioning teams improve patients’ outcome and working environment teamwork.*

## Discussion

This study offered insights into whether learning styles might affect attitudes to interprofessional learning among a sample of 34 nursing students. No statistical relationship could be established between learning styles and attitudes to interprofessional education which is in line with previous reported research [18]. Our results revealed that the accommodating learning style was the most frequent at the start of education. This overrepresentation of a concreate learning style in nursing students, using a pragmatic approach to guide the learning, based on practical application with a focus on feasibility, usability, and utility, corresponds well with previous research. [7, 31]. Practicality and usability correspond to instances in the interviews where students highlighted the ability to work in functional teams to ensure patient safety, improve patient outcome resulting in *increased quality of care*. It is important to bear in mind that Kolb’s LSI has sometimes have been misunderstood as describing static traits and not regarded as a dynamic states in the learning cycle process, supporting identification of learning style and thereby developing the ability to engage all modes of the learning cycle [32, 33]. However its aim is not to determine fixed learning traits [6] The LSI reflects the individuals’ perception of their way of learning at the particular time of the self-assessment. Nonetheless, students’ emphasis on the opportunity of interprofessional learning during internship and to work together with others were aspects brought to the fore in the interviews.

We found that nursing students welcomed studying and working together with other professions to develop *understanding of own profession* and a *deepened insight of care*. The positive attitudes towards interprofessional cooperation could be explained by the fact that nursing students are educated and trained to develop a holistic approach, including teamwork, to patient care. The positive attitudes to interprofessional cooperation may also be related to gender and the educational program. It has been found that female students in general, and nursing students in particular, are more open-minded about interprofessional cooperation compared to male students [34, 35]. Further, former hierarchical structures are nowadays experienced as relatively loose in Swedish healthcare. Students’ competence to identify comprehensively patients’ nursing needs, together with respect of the nursing competence of other professionals, might be an explanation for their positive attitude to teamwork. On the other hand, students clearly stated that a non-functional team had a negative effect on both patients’ outcome, and the working environment, *highlighting that it is amazing when it’s functional.*

## Conclusion

No statistic significant relationship was found between LSI and RIPLS. However, the nursing students underlined the importance of a successful teamwork. Both the universities and the organisers of the clinical settings need to make efforts to give students the opportunity to develop together with the interprofessional team. The transformative learning process is potentially useful to encourage deep learning in interprofessional settings. The purpose of the teacher-centred process transformative learning is to guide the student to acquire emotional and rational skills through reflection to develop new perspectives and meaning structures in line with the experiential learning cycle [4, 36]. Due to the interdependence of different professions in interprofessional teams a development of transformative learning settings may be a useful strategy for fostering interprofessional relationships [37, 38]. Further research is needed to explore useful strategies in how to educate students in using reflection and develop a critical professional mindset.

### Strengths and limitations

The findings presented link in interesting ways to the more general concept of authenticity and Mezirow’s theory of transformative learning [36, 39], which may potentially enhance the generalizability of the results. The same procedure and instruments for data collection were used on each occasion although students started their studies at different times. To enhance trustworthiness various measures were taken. For dependability, were all interviews were performed by the first author and the analysis was discussed in the research group until consensus; for credibility methodological triangulation was used; and for confirmability was students’ participation in the study was not discussed in educational settings to avoid influence from teachers [25]. Despite the low number of participants, the study could be considered to have informative power related to the purpose of the study, selection of students during ongoing education, the use of validated instruments and the analysis through mixed methods [40]. To maintain quality of the interviews all questions from the same interview guide was asked and all interviews were performed by the same person. [40]. It could be considered both a limitation and a strength that the interviewer had a pre-understanding working as a lecturer at the university [41]. A possible limitation is that the data were collected at one university in Sweden. However, since the Swedish nursing program is regulated by national guidelines [42], the results are likely to be relevant to similar programs. Contextual boundaries need to be taken into consideration in assessing the transferability of the results to other contexts. Nevertheless, our findings might be relevant to education of a similar kind since nursing education is regulated nationally as well as globally.

## Electronic supplementary material

Below is the link to the electronic supplementary material.

Supplementary Material 1

## Data Availability

The datasets generated and/or analysed during the current study are not publicly available due to possible personal details about the participants that might need to be anonymised but are available from the corresponding author on reasonable request.
